# Dimensional anhedonia and the adolescent brain: reward and aversion anticipation, effort and consummation

**DOI:** 10.1192/bjo.2019.68

**Published:** 2019-11-14

**Authors:** Ewelina Rzepa, Ciara McCabe

**Affiliations:** School of Psychology and Clinical Language Sciences, University of Reading, UK; Associate Professor of Neuroscience, School of Psychology and Clinical Language Sciences, University of Reading, UK

**Keywords:** Depressive disorders, imaging, adolescent, reward, anhedonia

## Abstract

**Background:**

Given the heterogeneity of depression the Research Domain Criteria Framework suggests a dimensional approach to understanding the nature of mental illness. Neural reward function has been suggested as underpinning the symptom of anhedonia in depression but how anhedonia is related to aversion processing is unclear.

**Aims:**

To assess how the dimensional experience of anhedonia and depression severity relate to reward and aversion processing in the human brain.

**Method:**

We examined adolescents and emerging adults (*n* = 84) in the age range 13–21 years. Using a dimensional approach we examined how anhedonia and depression related to physical effort to gain reward or avoid aversion and neural activity during the anticipation, motivation/effort and consummation of reward and aversion.

**Results:**

As anhedonia increased physical effort to gain reward decreased. As anhedonia increased neural activity decreased during effort to avoid in the precuneus and insula (trend) and increased in the caudate during aversive consummation. We found participants with depression symptoms invested less physical effort than controls and had blunted neural anticipation of reward and aversion in the precuneus, insula and prefrontal cortex and blunted neural activity during effort for reward in the putamen.

**Conclusions:**

We show for the first time that both physical effort and neural activity during effort correlate with anhedonia in adolescents and that amotivation might be a specific deficit of anhedonia irrespective of valence. Future work will assess if these neural mechanisms can be used to predict blunted approach and avoidance in adolescents at risk of depression.

## Anhedonia

It has been suggested that examining clinical symptoms, such as anhedonia, as a continuum across the spectrum may be more useful for identifying neurobiological signatures and risk markers of depression.^1^ Anhedonia, reduced interest and pleasure, is related to abnormalities in the brain's reward mechanisms and is suggested as a possible biomarker for depression.^2,3^ Adolescence is a crucial period that increases vulnerability to depression^4,5^ and low positive affect in adolescents is found to predict later depression.^6^ Further, anhedonia compared with irritability, has been described as a hallmark of adolescent depression as it is associated with greater illness severity, depression episodes, episode duration and suicidality.^7^

Interestingly, recent studies on the relationship between the experience of reward anticipation and active behaviour, in everyday life, find that depression symptoms weaken this relationship in young people.^8^ Taken together, anhedonia and reward processing have been clearly identified as important targets for treatment and prevention in adolescent depression.

## Anhedonia assessment

However, most studies assess anhedonia using only a few questions within other questionnaires such as the Beck Depression Inventory and do not provide a comprehensive understanding of the experience of anhedonia on a dimensional scale.^9^ Therefore we suggest using assessments such as the Temporal Experience of Pleasure Scales (TEPS) that allow for the separate components of anhedonia (anticipation and consummation) to be measured.^9,10^

Neurobiological studies have found blunted neural reward responses that relate to positive affect^11^ and depression symptoms in adolescents^12,13^ and even young children.^14^ However, most neurobiological tasks of reward do not examine the different phases of processing such as the anticipatory, motivational and consummatory aspects. This has led to inconsistencies across studies on reward in depression.^15^ For example how motivation for reward may relate to depression is rarely examined, which is interesting given that the construct of ‘diminished drive’ was better at predicting depression than the current DSM anhedonia criterion (that does not distinguish between anticipatory, motivational or consummatory aspects).^16,17^ Furthermore, recent behavioural data finds that adults with depression expend less effort for reward compared with healthy controls^18^ yet how this might be represented at the neural level is unknown. Therefore, to address this we have developed an experimental procedure that examines the anticipation of a food reward and a consummatory phase where rewarding food is eaten.

We have shown previously that those at risk of depression have decreased responses to anticipation and consummation (sight and taste of chocolate reward) in both ventral striatum and anterior cingulate cortex (ACC).^19^ In a follow-up study we examined young people (16–21 years) with a family history of depression but no personal experience of depression and found diminished neural responses in the orbitofrontal cortex (OFC) and the dorsal ACC to rewarding stimuli, sight and taste combined in the at-risk group.^20^ However these previous studies did not adequately separate the components of anticipation, motivation and consummation as trials included both taste and sight stimuli at the same time.

## Assessing anticipation, effort and consummation

Therefore, to address this we have extended our design to include after the anticipation phase and before the consummatory phase, an effort/motivational phase, to achieve reward and avoid aversive taste. We employed hard- and easy-effort phases so that we could have a roughly equal number of trials (*n* = 10) where reward and aversive taste was received and also to allow for the contrast between high and low effort. Using this, our recent study found that we could better separate the phases of reward processing in time.

We found that regions such as the medial prefrontal cortex (PFC), ACC and posterior cingulate cortex were activated to the cues, the insula and precuneus were activated during effort, and the caudate, ACC and insula were activated during consummation.^21^ However, when examining neural activity between young people with depression symptoms and controls and using a region-of-interest analysis we found activity in regions such as the pregenual ACC (pgACC) and ventral medial PFC (vmPFC) blunted across all reward and aversion phases in adolescents with depression symptoms, compared with controls.

Whole-brain analysis further revealed blunted activity in the precuneus and inferior frontal gyrus (during aversive anticipation) and hippocampus (during effort for reward) and ACC/frontal pole (during aversive consummation) in young people with depression symptoms. We also found a negative correlation between pgACC activity during reward consummation and anhedonia in adolescents with depression symptoms.^21^ This study was the first study to examine the separate phases of reward processing including a physical effort component inside the scanner, to gain reward and avoid aversion, in adolescents at high versus low risk of depression.

The results supported previous studies of blunted neural responses to reward but extended it by finding blunted responses to aversive stimuli and during the effort phase. This is in keeping with the meta-analysis and first quantitative review of emotional reactivity in depression that found consistent reductions in both positive and negative reactivity, which supported our previous study.^19,22^ Interestingly, we did not find brain differences between the groups in the ventral striatum, which is consistent with our previous study examining young people at familial risk of depression but no personal depression experiences^20^ nor did we find any relationship between anhedonia and the ventral striatum. This suggests that perhaps striatal differences (in this task) are only detectable after having experienced clinical depression and is thus a state rather than a trait marker of depression. However, the main criticism of this study was that we had only 16 participants in the high depression symptoms group and 17 in the healthy controls, which requires therefore that the results are interpreted with caution.

In this study we therefore aimed to repeat the procedure but including a larger number of adolescents and this time with a greater range of depression symptoms (including adolescents who were clinically depressed). We also extended our analysis and used, for the first time, a dimensional approach using multiple regression analyses to better unpick the relationship between symptoms such as anhedonia and the neural responses in our task.

## Summary

Taken together, in this study we assess how the dimensional experience of anhedonia and depression severity relate to both reward and aversion processing in the human brain. We hypothesise that anhedonia will negatively correlate with regions such as the prefrontal cortex (pgACC/vmPFC) during anticipation and the insula and precuneus during effort and the caudate, ACC and insula during consummation. We hypothesise this irrespective of the valence of a possible outcome (positive-reward versus negative-aversive event) based on ours and previous studies using a similar task in both those with and without depression symptoms, as described above.

## Method

### Participants

We recruited from the general population adolescents and young people (*n* = 84 between 13 and 21 years, mean 18.09, s.d. = 1.89) with a range of depression symptoms in line with the Research Domain Criteria (RDoC) approach.^1^ We did this by placing different adverts, for example an advert for young people with depression symptoms and an advert for young people with no explicit mention of depression. Some participants had a clinical depression diagnosis from their general practitioner, a psychologist or a psychiatrist (*n* = 27), some were on antidepressants (*n* = 14) and/or had a history of antidepressants (*n* = 6) (see supplementary Table S4 available at https://doi.org/10.1192/bjo.2019.68) and some had no depression symptoms (*n* = 41). We also included data from those (*n* = 16) who had high depression symptoms (measured with the Beck Depression Inventory (BDI)^23^ and the Mood and Feelings Questionnaire (MFQ)^24^) from our previous paper.^21^ Therefore the participants in this study had a range of depression symptoms as can be seen from Table 1. We used the Structured Clinical Interview for DSM-IV Axis I Disorders Schedule (SCID) to exclude for any other psychiatric history. We excluded pregnancy and any contraindications to magnetic resonance imaging (MRI).

**Table 1:** Demographics

	All (*n* = 84)	Depression symptoms group (*n* = 43)	Healthy control group (*n* = 41)	*P*
Age, years: mean (s.d.) range	18 (1.88) 13–21	18.16 (1.84)	18.02 (1.97)	0.74
Gender, female/male: *n*	64/20	33/10	31/10	0.904
Body mass index, mean (s.d.)	21.43 (2.34)	21.83 (2.18)	21.09 (2.41)	0.144
BDI, mean (s.d.)	16.81 (16.3)	29.88 (12.77)	3.32 (4.1)	<0.001
TEPS A, mean (s.d.)	41.98 (9.43)	36.28 (8.78)	48 (5.85)	<0.001
TEPS C, mean (s.d.)	33.62 (7.33)	30.35 (6.24)	36.78 (7.05)	<0.001
Chocolate, mean (s.d.)				
Craving	–	6.77 (2)	6.55 (2)	0.622
Liking	–	8.6 (1.33)	8.52 (1.37)	0.786
Frequency	–	2.67 (2.31)	2.43 (1.82)	0.588

BDI, Beck Depression Inventory; TEPS, Temporal Experience of Pleasure Scale; A, Anticipation; C, Consummation.

All procedures contributing to this work comply with the ethical standards of the relevant national and institutional committees on human experimentation and with the Helsinki Declaration of 1975, as revised in 2008. All procedures involving human patients were approved by The National South Central NHS ethics committee ref no: 14/SC/0102 and Reading University Research Ethics Committees and written informed consent was obtained.

### Questionnaires

The BDI measures the severity of depression from lack of depression to extreme clinical depression. The MFQ measures depression symptoms in adolescents and young people. On both of these scales greater depression severity results in a higher score. We examined TEPS^25^ – A, anticipatory and C, consummatory, where high scores indicate high anticipatory and consummatory pleasure thus low scores indicate anhedonia.

### Overall design

Participants were asked to refrain from consuming chocolate 24 h prior to scanning, in an effort to enhance the reward response for chocolate during the scan across participants and avoid increased satiety in some participants who may have a lot of chocolate before the scan. The task was adapted from McCabe *et al*^26^ to include an effort phase (supplementary Fig. S1). The task (40 trials) had four conditions based on the trial type (reward/aversive) and its effort level (easy/hard) this was to ensure that on reward-easy trials chocolate was received and on aversive-hard trials aversive taste was received. Trial type was cued by a visual stimulus (chocolate picture or a picture of a mouldy drink, 2 s, anticipatory phase), which indicated either to work to win the chocolate taste or to avoid the unpleasant taste. Effort was measured by the amount of button presses required to complete the effort phase (easy,  24; hard, 45 button presses). The effort phase, required volunteers to press a button as fast as possible (<6 s) to move a bar towards the pleasant chocolate picture (reward) and away from the unpleasant mouldy picture (aversive), allowing enough time to complete easy trials but not hard. A taste was then delivered (consummatory phase) based on performance. If on reward trials volunteers were successful they received chocolate taste (5 s delivery and 2 s swallow cue) and if not they received the tasteless solution. If on aversive trials volunteers were successful they received the tasteless solution and if not they received the unpleasant taste. A grey image (2 s) followed by a tasteless rinse was presented at the end of each trial. Each condition was repeated ten times, chosen by random permutation. Jitters were used for both interstimulus intervals and intertrial intervals. To sustain effort, four trials (two reward/two aversive) were longer at 9 s each. Volunteers also rated ‘wanting’, ‘pleasantness’ (+2 to –2) and ‘intensity’ (0 to +4) on a visual analogue scale on each trial (supplementary Fig. S1).

### Stimuli

The reward was a Belgian chocolate drink and the aversive was a combination of the same chocolate drink mixed with ‘Beet it’ beetroot juice, thus providing a similar texture but negative in valence. A tasteless solution (25 × 10^−3^ mol/L KCl and 2.5 × 10^−3^ mol/L NaHCO_3_ in distilled H_2_O) was also used as a rinse between trials. Solutions were delivered manually through three Teflon tubes allowing 0.5 mL to be delivered, similar to our previous studies.^27^

### Functional MRI scan

An event-related interleaved design and Siemens Magnetom Trio 3T whole-body MRI scanner and a 32-channel head coil were used. Multiband accelerated pulse sequencing (version no. RO12, Center for Magnetic Resonance Research, University of Minnesota, USA, EPI 2D BOLD/SE/DIFF Sequence) was used with an acceleration factor of six. *T*_2_*-weighted echo planner imaging slices were obtained every 0.7 s (repetition time). Fifty-four axial slices with in-plane resolution of 2.4 × 2.4 mm and between-plane spacing of 2.4 mm were attained. The matrix size was 96 × 96 and the field of view was 230 × 230 mm. Acquisition therefore was ~3500 volumes. An anatomical *T*_1_ volume with sagittal plane slice thickness of 1 mm and in-plane resolution of 1.0 × 1.0 mm was also acquired.

### Functional MRI analysis

Statistical Parametric Mapping (SPM)8 was used for realignment and normalisation of the images from echo planar imaging (EPI) to the EPI template of the Montreal Neurological Institute coordinate system and spatial smoothing with a 6-mm full-width-at-half-maximum Gaussian kernel. The time series at each voxel was low-pass filtered with a haemodynamic response kernel. Time series non-sphericity at each voxel was estimated and corrected for, with a high-pass filter with cut-off period of 128 s.

In the single-event design, a general linear model was then applied to the time course of activation in which stimulus onsets were modelled as single impulse response functions and then convolved with the canonical hemodynamic response function. Linear contrasts were defined to test specific effects. Time derivatives were included in the basis functions set. Following smoothness estimation, linear contrasts of parameter estimates were defined to test the specific effects of each condition (pleasant/unpleasant cue – grey image, pleasant/unpleasant taste – rinse, reward/aversive effort hard–effort easy) with each individual data-set. Voxel values for each contrast resulted in a statistical parametric map of the corresponding *t* statistic (transformed into the unit normal distribution (SPM *z*)). Movement parameters and parameters of no interest (such as the subjective ratings onset times) were added as additional regressors.

At the second level we examined the main effects of the task across all participants across the whole brain using one-sample *t*-tests, thresholded at *P*<0.05 corrected (familywise-error (FWE) (supplementary Table S5). Contrasts of interest were: anticipation phase: reward cue – grey control image and aversive cue – grey control image; effort phase: reward hard–reward easy and aversive hard–aversive easy; consummatory phase: reward taste–rinse (tasteless solution) and aversive taste–rinse (tasteless solution).

In line with a dimensional approach we examined the relationship between neural responses and the symptoms (depression and anhedonia) across all participants using a multiple regression analyses in SPM. For example, all participants' scans for the condition reward cue were entered into a model as a regressor with the corresponding participant's questionnaire data from the BDI (depression severity), the TEPS A and TEPS C (anticipatory and consummatory anhedonia) added as additional regressors. This allowed us to run correlations between neural activity and depression severity while controlling for anhedonia and vice versa. All analyses also had age, gender, history of medication and current medication added as covariates of no interest. For the effort conditions (for example reward hard–reward easy trials) the difference in time taken on each trial to complete effort-easy and effort-hard conditions (approx. 1 s difference) was also added as a covariate and the number of button presses per person per trial added as another covariate. We report multiple regression data thresholded at *P*<0.005 uncorrected and whole brain cluster corrected *P*<0.05 (FWE).

Using a categorical approach we also examined the difference in neural responses between those with depression symptoms and those with no symptoms using two-sample *t*-tests in SPM and thresholded at *P*<0.001. A total of 43 adolescents were deemed as having depression symptoms (depression symptoms group) as they had either a current diagnosis of major depression disorder (*n* = 27) from their general practitioner, clinical psychologist or psychiatrist or they scored >27 on the MFQ. There were 41 individuals regarded as having no depression symptoms (healthy control group) as they scored <15 on the MFQ and reported no symptoms during the SCID.

## Results

### Demographic data

Table 1 shows there were no group differences in age, gender, BMI, craving, liking and the frequency of chocolate eating between the depression symptoms group and the healthy control group. However, responses, on anhedonia and depression questionnaires differed between the groups as expected.

### Subjective ratings of stimuli: wanting, liking and intensity

We ran a mixed measures ANOVA with ratings as the first factor, three levels (wanting, pleasantness, intensity) and condition as the second factor, two levels (reward, aversive) and between-participant factor group (depression symptoms and healthy control groups). We found a significant main effect of ratings (*F*(1.1, 91.5) = 933; *P*_*GreenhouseGeisser-corrected*_ (*P*_*GG*_) < 0.001, η² = 0.91) and condition (*F*(1, 82) = 1537; *P*_*GG*_ < 0.001, η² = 0.94) but not group (*F*(1, 82) = 2.87; *P*_*GG*_ = 0.094, η² = 0.34). There was a significant interaction between rating and condition (*F*(1.3, 112) = 1097; *P*_*GG*_ < 0.001, η^2^ = 0.93) but no significant interaction between condition and group or between condition and group and ratings (all *P* > 0.1). *Post hoc* paired samples *t*-tests revealed that there was a significant difference in the wanting ratings for reward compared with aversion (*t*(83) = 53, *P* < 0.001, η² = 0.89) and in the pleasantness ratings of reward compared with aversion (*t*(83) = 41, *P* < 0.001, η² = 0.83) but no differences in the reported intensity of reward compared with aversion (*t*(83) = −1.57, *P* = 0.119, η² = 0.0009) (supplementary Table S1).

### Physical effort

**Fig. 1:**
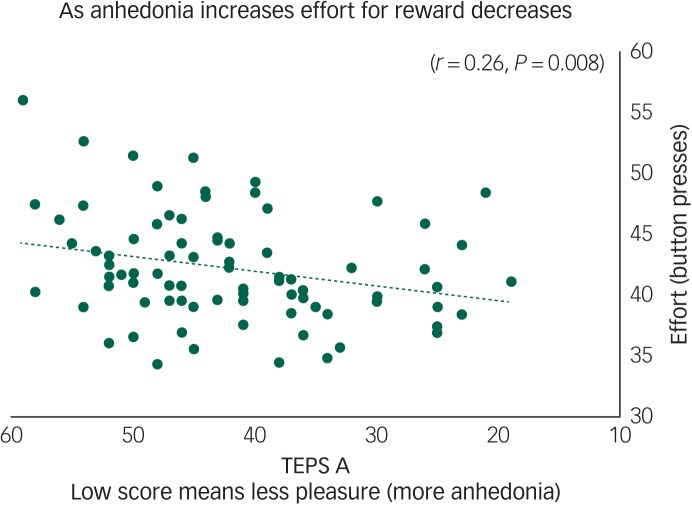
Anhedonia (Temporal Experience of Pleasure Scale, anticipation (TEPS A)) correlates with effort to gain reward on hard trials (*r* = 0.26, *P* = 0.008) across all participants.

Across all participants we found that anticipatory pleasure (TEPS A) positively correlated with effort on hard reward trials (*r* = 0.26, *P* = 0.008) (Fig. 1). The TEPS is a measure of pleasure, thus a low score indicates anhedonia. Figure 1 shows that as effort decreases pleasure also decreases (anhedonia increases). There were no significant correlations with the TEPS C.

Using a mixed-measures ANOVA with effort (number of button presses) as the first factor, two levels (hard and easy) and condition as the second factor, two levels (reward, aversive) and between-participant factor of depression symptoms and controls we found a significant main effect of effort (*F*(1, 82) = 493; *P*<0.001, η² = 0.85) and of condition *F*(1, 82) = 14.1; *P*<0.001, η² = 0.15) and a significant main effect of group (*F*(1, 82) = 5.49; *P* = 0.02, η² = 0.063). There was also an effort by condition interaction (*F*(1, 82) = 23; *P* < 0.001, η² = 0.22) but no significant interaction between condition and group or between condition, group and effort (all *P* > 0.1). *Post hoc* paired samples *t*-tests revealed that participants expended greater effort to gain reward on the hard versus easy trials (*t*(83) = −20, *P* < 0.001, η² = 0.55) and to avoid aversion on the hard versus easy trials (*t*(83) = −21.1, *P*<0.001, η² = 0.57). Follow-up one-way ANOVA revealed that the healthy control group expended more effort on the reward-hard trials than the depression symptoms group (*F*(1, 82) = 4.5; *P* = 0.037, η² = 0.47) but there were no group differences on the reward-easy trials or the aversive trials (all *P*>0.1) (supplementary Table S3).

### Exploratory gender differences

Although the data were skewed in favour of female participants we did some preliminary exploratory gender analyses. There were no gender differences in age, BDI, chocolate craving, liking or frequency of eating. We examined the effect of gender on anhedonia and used a mixed-measures ANOVA with TEPS, two levels (TEPS A and TEPS C) and between-participant factor of gender (male and female participants). We found a significant main effect of TEPS (*F*(1, 82) = 62; *P*_*GG*_ < 0.001, η² = 0.43) and of gender (*F*(1, 82) = 4.4, *P* = 0.03, η² = 0.05). A follow-up one-way ANOVA revealed that boys had greater consummatory anhedonia (*F*(1, 82) = 6.6, *P* = 0.01, η² = 0.08) compared with girls.

To examine effort we used a mixed-measures ANOVA with effort as the first factor, two levels (hard and easy) and condition as the second factor, two levels (reward, aversive) and between-participant factor of gender (male and female participants) we found a significant main effect of effort (*F*(1, 82) = 436; *P*_*GG*_ < 0.001, η² = 0.84) and of condition (*F*(1, 82) = 8.9; *P*_*GG*_ = 0.004, η^2^ = 0.09) and a significant main effect of gender (*F*(1, 82) = 3.5; *P* = 0.05, η² = 0.04). There was also an interaction between effort and condition (*F*(1, 82) = 16.8; *P*_*GG*_ < 0.001, η² = 0.16) and effort and gender (*F*(1, 82) = 7.2; *P*_*GG*_ = 0.009, η² = 0.08). Follow-up one-way ANOVA of the four conditions, reward-easy, reward-hard, aversive-easy, averersive-hard trials found that girls made significantly less effort for reward-hard trials than boys (*F*(1,82) = 4.9; *P*_*GG*_ = 0.029, η² = 0.06).

Taken together, although there were many more female than male participants in this study these results suggest possible gender differences in anticipatory and consummatory anhedonia and highlight the need for studies directly examining gender effects on symptoms like anhedonia.

## Functional MRI results

### Main effect of task

The main effect of task used one-sample *t*-tests in all participants (supplementary Table S5).

#### Anticipation phase

Cues for both reward and aversion activated regions such as the occipital lobes, but also the pgACC/vmPFC and parts of the superior and middle frontal gyrus. The aversive cue, however, also activated the parietal lobe.

#### Effort phase

Achieving rewards and avoiding aversion activated the insula, motor and premotor areas whereas the putamen and parietal lobe were activated for the effort for reward.

#### Consummation phase

Both tastes activated dorsal ACC regions with the middle OFC also activated to the taste of reward. The caudate was also activated by the aversive taste.

### Dimensional anhedonia and depression severity correlations with neural responses

See Table 2 for dimensional anhedonia and depression severity correlations with neural responses.

**Table 2:** Multiple regression results: relationship between neural responses to reward and aversion and depression severity and anhedonia symptoms^a^

Brain region	Comment	*X*	*Z*	*Y*	*z*	*P*	*β*	*f*^2^
Anticipation phase	No significant correlations							
Effort phase	Positive correlation between effort to avoid aversion (aversive hard–aversive easy) and anticipatory anhedonia (TEPS A)							
Precuneus (BA7)		4	−56	46	3.67	0.01^b^	0.06	0.93
Insula		−36	−6	2	4.19	0.06^c^	0.06	0.91
Consummation phase	Negative correlation between aversive taste and consummatory anhedonia (TEPS C)							
Caudate		−14	−4	26	4.39	0.006	−0.09	0.83

TEPS, Temporal Experience of Pleasure Scale; A, Anticipation; C, Consummation.

a. For the effort conditions results remained the same when number of button presses were added as covariates of no interest. Beta values were extracted from SPM and *f*^2^-values were calculated by examining the brain data from plots in excel and running a correlation against the symptom questionnaires to get the *r* values.

b. Becomes non-significant when age is removed as a covariate.

c. Becomes more significant when age removed as a covariate.

#### Anticipation phase

We found no significant correlations between the anticipation phases and depression severity or anhedonia across all participants.

#### Effort phase

**Fig. 2:**
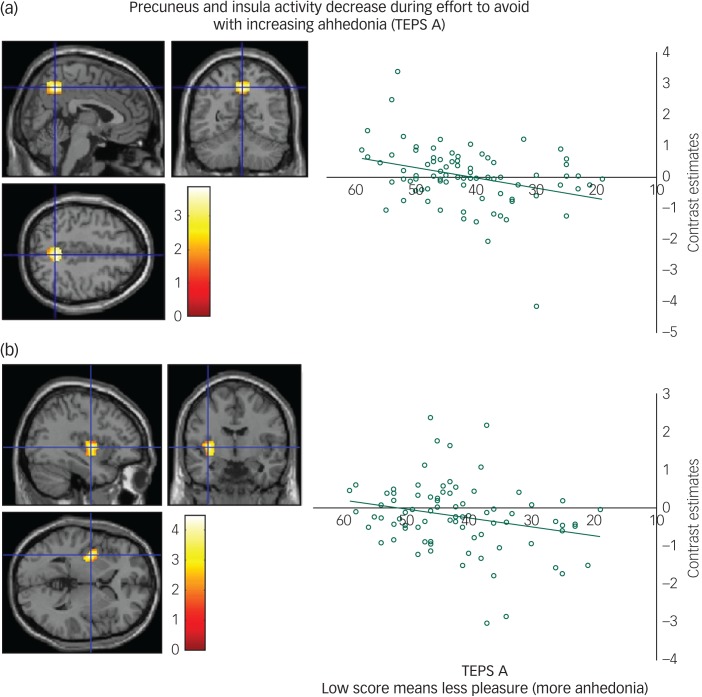
(a) Precuneus activity during effort to avoid correlates with anticipatory anhedonia: left panel, axial, sagittal and coronal image (4, −56, 46) *z* = 3.67 *P* = 0.01; right panel, contrast estimates for precuneus correlated with anhedonia (Temporal Experience of Pleasure Scale, anticipation (TEPS A)). (b) Insula activity during effort to avoid correlates with anticipatory anhedonia: left panel, axial, sagittal and coronal image (−36, −6, 2) *z* = 4.19; *P* < 0.06 right panel, contrast estimates for insula correlated with anhedonia (TEPS A).

We found a positive correlation between the TEPS A anticipatory anhedonia questionnaire and effort to avoid aversion in the precuneus (Fig. 2(a)) and a trend also in the insula (Fig. 2(b)), meaning that as ability to anticipate pleasure decreased so did activity in these regions.

#### Consummation phase

**Fig. 3:**
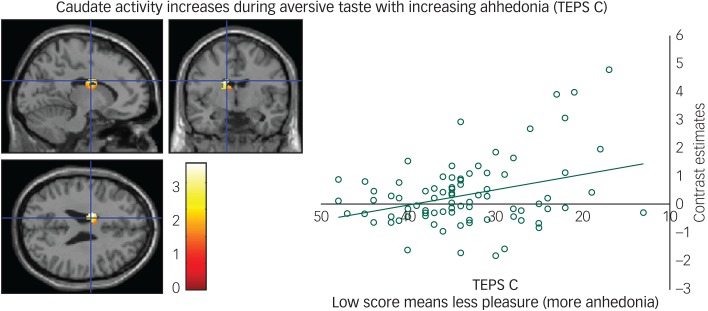
Caudate activity during aversive taste correlates with consummatory anhedonia: left panel, axial, sagittal and coronal image (−14, −4, 26) *z* = 4.39, *P* = 0.006); right panel, contrast estimates for caudate correlated with anhedonia (Temporal Experience of Pleasure Scale, consummation (TEPS C)).

We found a negative correlation between the TEPS C consummatory anhedonia questionnaire and the neural response during the aversive taste in the caudate (Fig. 3), meaning that as ability to experience pleasure decreased, activity in the caudate increased.

### High depression symptoms versus low depression symptoms

#### Anticipation phase

We found decreased activity in the depression symptoms group during the anticipation of both reward and aversion in the precuneus, insula, lateral OFC and dorsal lateral PFC (dlPFC) compared with controls (Table 3). We also found decreased activity in the depression symptoms group during the anticipation of aversion in the premotor cortex and posterior cingulate, compared with controls.

**Table 3:** Between-group results: high depression symptoms versus healthy control group analysis^a^

Brain region	Comment	*X*	*Y*	*Z*	*z*	*P*	Standard effect size	Upper 95% CI	Lower 95% CI
*Anticipation phase*									
Chocolate picture									
Healthy controls versus depression symptoms group									
lOFC (BA10)/		−38	48	−4	5.39	<0.001	−0.57	2.12	0.23
dlPFC (BA9)		−28	36	30	5.39	<0.001	−0.85	1.08	−0.24
Insula		42	6	0	4.78	<0.001	−0.92	0.37	−0.88
Precuneus (BA7)		10	−70	48	4.71	<0.001	−2.26	0.17	−0.84
Aversive picture									
Healthy controls versus depression symptoms group									
lOFC (BA10)/		−38	48	−2	5.05	<0.001	−0.82	2.57	0.44
dlPFC (BA9)		−30	44	30	5.05	<0.001	−0.72	2.25	0.4
Precuneus (BA7)		−2	−74	−46	4.11	0.009	−0.84	0.06	−1.24
Insula (BA13)		30	22	14	4.09	0.01	−0.77	1.09	−0.4
Premotor cortex (BA6)		38	12	42	4.01	0.01	−0.64	1.04	−0.08
Posterior cingulate (BA31)		8	−30	42	3.93	0.01	−0.90	1.08	0.26
*Effort phase*									
Effort to gain chocolate (choc hard-choc easy)									
Healthy controls versus depression symptoms group									
Insula		32	16	−2	3.76	0.005	−0.61	0.41	−0.002
Claustrum/putamen		26	8	−4	3.45	0.005	−0.58	0.33	−0.09
Effort to avoid aversion (aversive hard-aversive easy)	No significant differences								
*Consummation phase*									
Chocolate taste or aversive taste	No significant differences								

CI, confidence interval; lOFC, lateral orbitofrontal cortex; dlPFC, dorsal lateral prefrontal cortex.

a. Thresholded at *P* = 0.001. Standard effect size calculated using the depression symptoms and healthy control groups extracted activation from SPM plots. The mean activation for each group and the s.d. and *n* was then used in the Centre for Evaluation and Monitoring online effect size calculator.

#### Effort phase

**Fig. 4:**
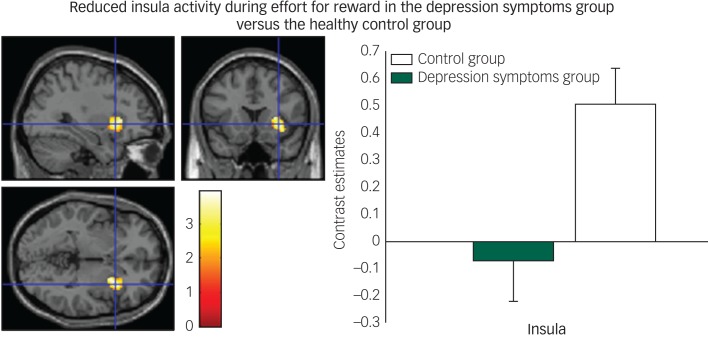
Reduced insula activity in depression symptoms group versus controls during effort for reward: left panel, axial, sagittal and coronal image (32, 16, −2) *z* = 3.76, *P* = 0.005); right panel, contrast estimates for insula for depression symptom and control group.

We found decreased activity in the depression symptoms group during the effort to gain reward in the insula (Fig. 4) and claustrum/putamen compared with controls.

#### Consummation phase

We found no significant group differences for the consummatory phase. Also we did not find any regions increased, in any phases, in the depression symptoms group compared with the healthy control group.

## Discussion

In line with the National Institute of Mental Health RDoC initiative we examined how the dimensional symptoms of anhedonia mapped onto the neurobiology of reward and aversion processing in adolescents and young people with a range of depression symptoms. We examined how dimensional anhedonia, as measured by the TEPS, related to the anticipation (sight) and consummation (taste) of both reward and aversion. As it has been shown that the construct of ‘diminished drive’ was a better predictor of depression than the DSM anhedonia criterion^16,17^ we also introduced an effort phase as a way of measuring motivation and drive similar to that used in preclinical work.^28^

### Effort for reward and aversion correlates with anhedonia

We found that anticipatory anhedonia increased as effort (button presses) to gain reward decreased across all participants. This is interesting in that it shows how the experience of anhedonia is related to physical motivation/effort for reward in adolescents. Across all participants we also found that decreased precuneus and insula activity during effort to avoid aversion correlated with anticipatory anhedonia. This is interesting as it is the first evidence showing that anhedonia is related to reduced neural activity during effort to avoid, suggesting that anhedonia is underpinned not only by a deficit in reward, as widely reported, but also by a neural deficit during the avoidance of unpleasant situations too. This suggests that motivation might be an important component of anhedonia irrespective of valence, which is also in keeping with the meta-analysis finding that responses to both reward and aversive events are blunted in depression.^29^ This also fits with our previous work that finds blunted responses to reward and aversion in young people with depression symptoms.^21^

The precuneus, situated in the posteromedial portion of the parietal lobe, has been associated with self-related mental representations in those with and without depression.^30,31^ Furthermore a meta-analysis identified increased activation in the parietal lobule (for example superior parietal gyrus, inferior parietal lobule) in response to negative stimuli in major depressive disorder^32^ and resting-state studies suggest activity in the precuneus related to excessive self-focus (for example rumination) in depression.^33,34^ Taken together, reduced precuneus activity during effort to avoid might be a mechanism by which blunted self-focus reduces sensitivity to how negative stimuli might affect oneself and therefore in turn increases exposure to negative events.

We also found a trend for decreased insula activity and anticipatory anhedonia during effort to avoid. The insula has been implicated in many studies of depression examining functional connectivity, volumetric analyses and biomarkers for treatment outcomes.^35^ The insula is also involved in emotion processing, self-awareness and motor control^36^ and also specifically in anticipatory cues and approach and avoidance behaviour.^37,38^

Future longitudinal studies should directly assess if activity in the precuneus and insula can be used to predict changes in avoidance behaviour in those at risk of clinical depression. Interestingly a recent study found insula hypermetabolism was associated with treatment response in depression, specifically remission to escitalopram.^39^

Furthermore, and consistent with our previous studies, we found no relationship between anhedonia and ventral striatal activity in our task^40^ again suggesting that ventral striatal deficits might be a state rather than trait marker of anhedonia. We also found that as consummatory anhedonia increased, the neural response during aversive taste increased in the caudate. Consistent with this, we have found in previous studies, using similar stimuli, increased response in the caudate to aversive taste in those recovered from depression.^19^ The caudate receives dopaminergic projections and its activity in relation to reward processing is well established, as it receives inputs from motor areas and those involved in affective processing, such as the vmPFC, amygdala and insula.^41^ Although, more recently, Carretie *et al* report the caudate responding strongly to negative stimuli above and beyond its response to positive.^41^ Further, this effect was not explained by differences in arousal levels and led Carretie *et al* to claim support for models proposing the striatum as a key element in withdrawal situations.^41^ Extending this, our results show that as the caudate increases in activity to aversion, consummatory anhedonia also increases, therefore it is possible that increased caudate activity could be one mechanism by which overprocessing of negative information prevents in-the-moment enjoyment and pleasure.

### Between-group differences

Using a categorical approach we also found participants with depression symptoms invested less physical effort to gain chocolate reward than those with no symptoms. This is consistent with previous literature finding reduced effort for reward in depression^42,43^ but extends the findings to primary reward in adolescents. Supporting our dimensional results we also found decreased precuneus and insula activity in those with depression symptoms, compared with controls, during the anticipation of reward and aversion. This is thus consistent with our aforementioned suggestion that blunted activity in these regions might be a mechanism by which imagining future events and how they relate to the self is difficult and thus could limit how people with depression prepare to gain positive or avoid negative situations. Future studies should directly examine how imagery about behavioural outcomes related to the self affect activity in these regions.

We also found reduced activity in the left dlPFC and lateral OFC during anticipation for reward and aversion in the depression symptoms group. The dlPFC is involved in executive function and cognitive control over behaviour and action.^44^ The dlPFC has been found to have functional and structural asymmetry that correlates with depressive symptoms from healthy young adults to individuals with subclinical depression and patients with major depressive disorder, using an electroencephalogram. Previous studies report that the left dlPFC is hypoactive for positive and the right dlPFC hyperactive for negative stimuli^45,46^ yet we found blunted left dlPFC to the anticipation of both positive and negative in our study. This might indicate further a mechanism by which reduced planning to gain reward and avoid aversion might arise in those with depression symptoms.

The lateral OFC connects to the dlPFC, insula and premotor areas^47^ and has been found to have a critical role in reversal learning and adapting behaviour based on the most rewarding outcome.^48^ Interestingly, it has been suggested that dysfunction of the lateral OFC ‘non-reward’ circuit may lead to the generation of negative self-thoughts and reduced self-esteem apparent in depression.^47,49^ Therefore reduced lateral OFC activity in this study might indicate a mechanism by which those with depression symptoms are less able to switch their behaviour in preparation to gain reward or avoid aversion.

We also found reduced activity during effort to gain reward in the putamen in the depression symptoms group. The putamen is involved in anticipation and instrumental action^50^ and has been found deficient in connectivity with the medial OFC in depression, which appears to underlie the dysfunction of effort-based valuation processing.^51^ Further in the same study the authors report that greater amotivation severity was found to correlate with smaller work-related putamen activity changes for reward in schizophrenia and effort in depression.^51^ Therefore our result is consistent with abnormal functioning of the putamen in depression in relation to effort processes but extends this for the first time to adolescents and natural primary reinforcers.

### Future directions

Exploratory analysis (as we had more female than male participants) revealed boys had more consummatory anhedonia than girls whereas girls made less effort for the reward-hard condition than boys. These preliminary results suggest there may be differences between girls and boys in different components of anhedonia (anticipation and motivation and consummation). Although our data were skewed as we had more female than male participants our results are at least in line with the general population rates i.e. more female patients report depression than male patients; however, future studies would benefit from a comprehensive examination of gender effects. Given that girls experience depression approximately twice as much as boys it is of course imperative that we begin to examine gender differences so that personalised treatments can be developed.

Finally, it would be of interest to examine with a longitudinal study how the neural activity we identified, as related to anticipatory and consummatory anhedonia, can be used to predict blunted behavioural approach and avoidance in adolescents who are depressed and transdiagnostically across other adolescent disorders. Further it would be of interest to examine if there are specific age ranges when motivational behaviour and/or neural activity, are best at predicting future symptom change or onset. In turn, the aim would be to determine if we could use these neural responses as early markers of risk and markers of symptom improvement and treatment response.

## Supplementary material

For supplementary material accompanying this paper visit http://dx.doi.org/10.1192/bjo.2019.68. click here to view supplementary material
