# Supplementary material for: Dimensional anhedonia and the adolescent brain: reward and aversion anticipation, effort and consummation
Source: BJPsych Open. 2019 Nov 14;5(6):e99. doi: 10.1192/bjo.2019.68 (PMC6949536; doi:10.1192/bjo.2019.68)
Supplement: Supplementary file 1 [file S2056472419000681sup001.docx]

**Dimensional Anhedonia and the Adolescent brain: Reward and Aversion Anticipation, Effort and Consummation.**

**Short title**:

Anhedonia in adolescent depression

15. Swallow cue (2 sec)

14. Rinse delivered (5 sec)

**Figure S1.** Visual depiction of a reward-hard trial.


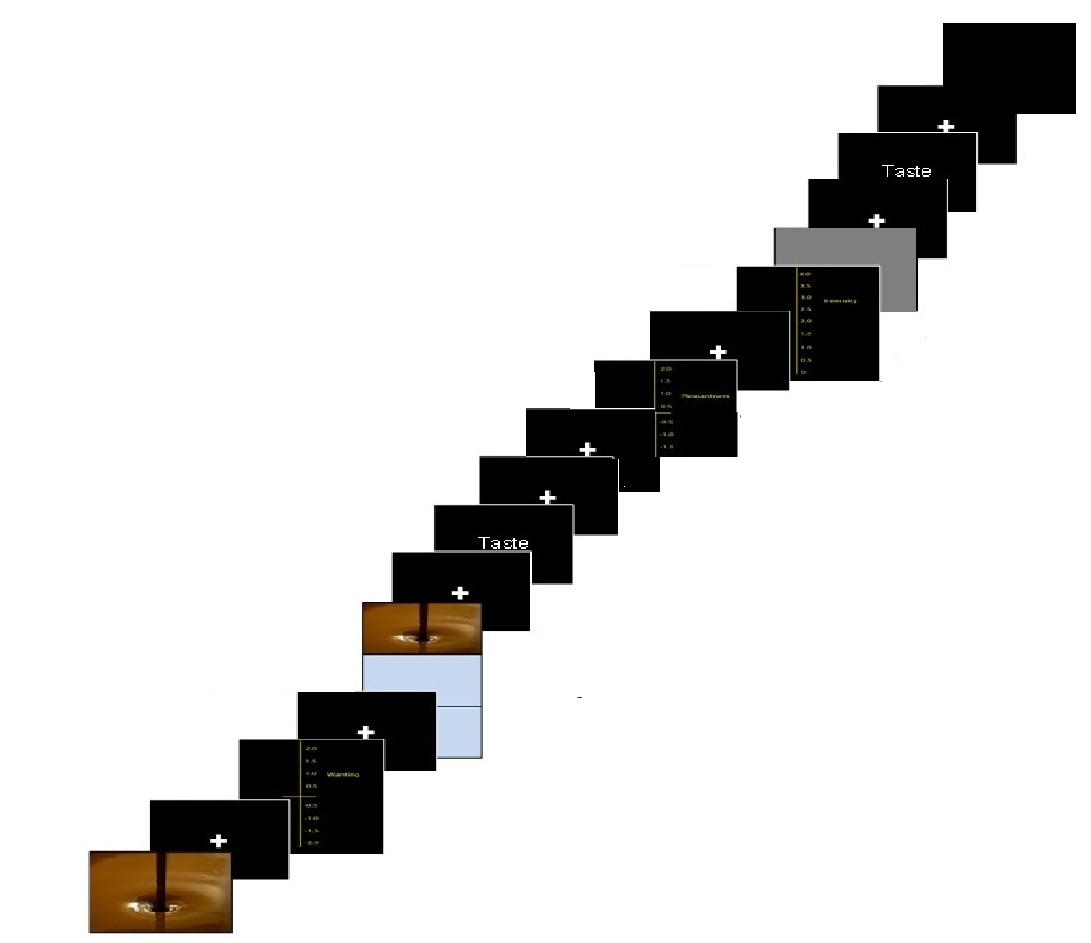


16. Blank (0.8-1.5 sec jittered)

15. Rinse (5 sec)

1. Cue (2 sec)

2. Fixation (1-3 sec jittered)

3. Wanting (5 sec)

4. Fixation (1-3 sec jittered)

5. Effort (6 sec)

7. Command cue (2 sec)

8. Taste (5 sec)

9. Swallow (3-5 sec jittered)

10. Pleasantness (5 sec)

11. Fixation (1-3 sec jittered)

11. Intensity (5 sec)

12. Grey image (2 sec)

13. Fixation (1-3 sec jittered)

14. Command cue (2 sec)

6. Fixation (1-3 sec jittered)

Table S1: Subjective ratings for all participants.

|  | Chocolate  mean (SD) | Aversive  mean (SD) |
| --- | --- | --- |
| Wanting | 1.52 (.38) | -1.7 (.29) |
| Liking | 1.47 (.38) | -1.54 (.49) |
| Intensity | 2.32 (.71) | 2.44 (.8) |

Table S2: Subjective ratings for DS and HC groups.

|  | Group | |  | |  | |
| --- | --- | --- | --- | --- | --- | --- |
| Ratings | DS | HC | DS | HC | DS | HC |
|  | Chocolate | | Aversive | | Tasteless solution | |
| Picture wanting; mean (SD) | 1.43 (.45) 1.62 (.25) | | -1.75 (.33) -1.75 (.25) | |  | |
| Taste liking; mean (SD) | 1.38 (.44) 1.56 (.28) | | -1.52 (.47) -1.55 (.48) | | .67 (.9) .57 (.85) | |
| Taste intensity; mean (SD) | 2.28 (.77) 2.37 (.63) | | 2.38 (.85) 2.51 (.74) | | 1.25 (.87) 1.53 (1) | |

Table S3: Number of button presses for effort phase for DS and HC groups.

|  | DS; mean (SD) | HC; mean (SD) |  |  |
| --- | --- | --- | --- | --- |
| *Button presses* |  |  |  |  |
| Reward Easy | 31.77 (1.27) | 32 (1.1) |  |  |
| Reward Hard | 41.25 (4.13) | 43.33 (4.44) |  |  |
| Aversive Easy | 31.42 (1.51) | 31.81 (.91) |  |  |
| Aversive Hard | 42.93 (4.67) | 44.48 (4.69) |  |  |
|  |  |  |  |  |
|  |  |  |  |  |
|  |  |  |  |  |
|  |  |  |  |  |
|  |  |  |  |  |

Table S4: Medication status of participants with MDD diagnosis.

| **Participant ID** | **Medicated during study** | **History of medications** |
| --- | --- | --- |
| 1 | Citalopram 10mg | Sertraline |
| 2 | - | Bupropion, Fluoxetine, Sertraline, Mirtazapine |
| 3 | Fluoxetine 50mg | - |
| 4 | Sertraline 100mg |  |
| 5 | - | Fluoxetine 20-60mg, Sertraline 50mg |
| 6 | Sertraline 100mg | Citalopram |
| 7 | Sertraline | - |
| 8 | - | Fluoxetine 20mg |
| 9 | Citalopram 20mg | - |
| 10 | Citalopram 40mg | - |
| 11 | - | Citalopram 20mg |
| 12 | Citalopram 30mg | - |
| 13 | Fluoxetine 40mg | - |
| 14 | Sertraline 10mg | - |
| 15 | Fluoxetine 60mg | - |
| 16 | - | Fluoxetine 20mg |
| 17 | - | Citalopram 20mg |
| 18 | Paroxetine 20mg | - |
| 19 | - | Citalopram 20mg |
| 20 | - | Fluoxetine 20mg |
| 21 | Citalopram 40mg | - |
| 22 | Sertraline 50mg | - |

Table S5: Regions showing whole brain responses to each phase in all participants.

| Whole Brain MNI coordinates | | | | | |
| --- | --- | --- | --- | --- | --- |
| Brain region | X | Y | Z | z-value | p-value |
| **Anticipation** | | | | | |
| ***Reward cue***  Occipital lobe (BA18)  Occipital lobe (BA18)  SFG (BA8)  MFG (BA10)  pgACC/vmPFC  pgACC/vmPFC  ***Aversive cue***  Occipital lobe (BA17)  Occipital lobe (BA18)  pgACC/vmPFC  SFG (BA8)  Parietal lobe (BA40)  SFG (BA9)  MFG (BA10)  **Effort**  ***Gain Reward: Choc hard-choc easy***  Primary motor (BA4)  Insula (BA13)  MFG premotor (BA6)  Parietal (BA40)  Putamen  SFG (BA9)  Insula (BA13)  ***Avoid Aversion: Aver hard-Aver easy***  Primary motor (BA4)  Occipital lobe (BA18)  MFG premotor (BA6)  Insula (BA13)  Primary motor (BA4)  Insula (BA13)  SFG (BA9)  **Consummation**  ***Chocolate Taste***  dACC (BA33/24)  Mid OFC (BA11)  ACC (BA24)  ***Aversive taste****  Caudate  MFG premotor (BA6)  dACC (BA32) | -16  18  14  34  -14  6  16  -16  10  14  60  -28  34  -32  -42  -4  56  -28  26  40  -34  -26  0  -48  28  -38  34  2  22  0  16  0  -2 | -94  -92  46  54  50  48  -86  -94  48  46  -44  54  54  -24  -26  -22  -32  0  56  6  -26  -92  -10  -22  -24  -20  50  12  30  28  18  14  32 | 8  6  52  4  2  4  -2  8  2  52  34  30  4  50  20  48  22  -4  30  0  58  2  56  18  56  -6  32  24  -12  20  12  42  20 | 10.33  9.51  7.48  5.38  5.29  4.93  7.1  7  5.74  5.67  5.41  5.31  5.13  7.68  7.68  6.2  5.43  5.28  5.16  5  7.57  7.48  6.32  6.10  5.74  5.33  5.29  4.9  4.92  4.84  3.82  3.88  3.07 | <0.001  <0.001  <0.001  =0.001  =0.002  =0.005  <0.001  <0.001  <0.001  <0.001  <0.001  =0.004  =0.003  <0.001  <0.001  <0.001  <0.001  <0.001  <0.001  <0.001  <0.001  <0.001  <0.001  <0.001  <0.001  <0.001  =0.008  <0.001  <0.001  =0.006  =0.002  =0.002  =0.002 |

---------------------------------------------------------------------------------------------------------------------

Thresholded at p<0.05 FWE whole brain corrected. Clusters corrected p<0.05 FWE.

* Thresholded at p=0.01 uncorrected. OFC- orbitofrontal cortex; ACC- anterior cingulate cortex; MFG- middle frontal Gyrus; SFG- superior frontal gyrus.
